# Moving toward a spiritual pedagogy in L2 education: Research, practice, and applications

**DOI:** 10.3389/fpsyg.2022.978054

**Published:** 2022-10-19

**Authors:** Dongmei Song

**Affiliations:** School of Foreign Languages, Xinyang Normal University, Xinyang, China

**Keywords:** L2 education, positive psychology, EFL student, spiritual pedagogy, EFL teacher

## Abstract

Spiritual pedagogy (SP) as a new educational theory aims to apply cultural and spiritual values in classroom practices. It has been the focus of research in different fields such as counselling, management, and science in the past decades. However, its application in second/foreign language research and practice has been widely overlooked by L2 researchers, to date. To fill this gap, the present study made an effort to provide a theoretical analysis of the conceptualizations, scientific background, benefits, and practical techniques to implement SP in the context of L2 education. In so doing, this paper suggests a number of practices through which SP can be integrated into L2 education among which positive psychology (PP) was more highlighted. Additionally, some practical implications were provided for EFL teachers, teacher trainers, students, curriculum designers, and researchers to enhance their knowledge of spirituality and education. In the end, different suggestions for future research were presented to move this research domain forward.

## Introduction

Teaching has long been considered as a complicated profession given the presence of an interplay of many psych-emotional, linguistic, and socio-cultural factors (Agudo, 2018; Benevene et al., 2020; Mercer, 2020). Each of these factors places a heavy pressure on teachers imposing them to be intellectually, physically, and emotionally strong (Davaribina and Asl, 2021; Li, 2021; Liu et al., 2021). However, as contended by the proponents of humanistic psychology and positive psychology (PP), emotional factors play a critical role in determining the success of failure of every aspect of education including teachers’ and students’ academic performance, satisfaction, classroom behavior, and practices (Shao and Parkinson, 2021; Sikma, 2021). Trying to go beyond negative emotions, PP capitalized on the power and significance of positive emotions in academia and personal/professional development (MacIntyre and Mercer, 2014; Dewaele et al., 2019; MacIntyre et al., 2020; Gregersen and MacIntyre, 2021; Wang et al., 2021; Xie and Derakhshan, 2021). Such a movement in educational psychology coupled with “education of the heart” facilitated the ground for uniting personal emotions to education and establishing a caring, mutual relationship among stakeholders in academic domains (Glazer, 1999; Noddings, 2002; Parmenter, 2006). These attempts to maintain and enhance care in education led to the introduction of a new concept in educational psychology called “spirituality” which calls for forging mutual associations among academic staff beyond ego (Noddings, 2002). Spirituality is considered as one of the cornerstones of education and social work (Wiersma, 2004; Kernochan et al., 2007) that includes values like teacher-student prudence, compassion, and sincerity and brings about efficiency and immediacy in education.

Due to its complications, the concept of spirituality has been given different definitions by different scholars (Harlos, 2000; Watson, 2014), yet the most common element in such definitions is “values.” According to Principe (1983), spirituality concerns how a person understands and lives the noblest values and works toward their perfection or fulfillment. Therefore, spirituality requires uniformity between one’s daily practices and his/her fundamental values (Hyde, 2005). After its application in religious domains, science, management education, and organizational research, spiritual pedagogy (SP, hereafter) positioned itself as a teaching approach in Western countries in the past decades (Masbur and Syahril, 2021). SP is a theory that intends to apply cultural and spiritual values to education (Shimabukuro, 2008; Masbur and Syahril, 2021). To put it differently, SP is an experiential, thoughtful, holistic, and reflective practice that considers the whole of a matter, while also attending to the importance of each element (Pettersen, 2015).

In teaching, spiritual values are driven from one’s experience obtained by pedagogical reflections, discussions, and indoctrination. SP approach was welcomed in educational research and practice due to the fact that every aspect of teaching is a part of spiritual reality and every moment in the classroom is a spiritual opportunity for teachers and students (Miller and Athan, 2007). Based on this approach, the classroom is by nature a spiritual space in which the teacher is not the only agent of generating learning and highlights the role of spiritual factors (Miller and Athan, 2007). Research approves that the integration of spirituality is effective in many professions (Bartoli, 2007) demanding individuals to be competent in term of understanding and dealing with spiritual variables at their work. Although research on various aspects of second/foreign language education is rapidly growing, the role of spirit and spirituality in L2 teachers’ and students’ practices has been widely under-researched, to date (Mahboob and Courtney, 2018). This runs counter to the very nature of teaching as a caring job in that jobs having to do to with people must consider the world of spirit as an element (Bradley, 2011). Most of the existing studies in this line of research have focused on the conceptualization of SP and its differences from religiosity. The empirical studies, also, have been limited to one-shot correlational studies to endorse the impact of SP on several constructs in education. Nonetheless, the research and practice of SP in the context of L2 education is left uncharted, to date. To fill this gap, the present article was an effort to review the related theories and conceptualizations behind SP and its scientific backgrounds and possible applications in the context of English language teaching (ELT) in L2 contexts.

## Background

### The conceptualizations of spirituality

Attempts to make the muddy water of defining the construct of spirituality clear has been the focal point of different studies. The outcome of such studies has been the proposition of numerous definitions and conceptualizations for the concept of spirituality (Harlos, 2000). According to Syihabuddin (2017), spirituality is a person’s conscious experience of connecting actual life with noble values. It is an everlasting value that leads human life making a specific value the core motive and trigger for doing certain activities (Syihabuddin, 2017). Although the term has been confused with religion and morality, in education it refers to a profound association between students, teachers, and the subject matter that is characterized by honesty, vitality, and excitement (Jones, 2005). In the existing body of research, the conceptualizations of spirituality can be categorized into “what spirituality is,” and “what spirituality is not” (Jones, 2005). To be more specific, the second classification considers “spirituality” as a mega concept that encompasses *transcendence* (moving beyond one’s psychological walls to experience the nature of things more deeply), *connection* (the bonds among teachers, students, and academic staff), *wholeness* (everything is already connected, in relationship, and in union), and *compassion* (sensitivity to others’ emotional states and their suffering) (Glazer, 1999; Nash, 2002). With respect to the third classification of defining spirituality, research substantiates that the concept is not equated with “religion, faith, ethics, doctrine, nor is it an individual trait” (Wolf, 1996; Nash, 2002; Dunne, 2003; Jones, 2005). However, these diverging conceptualizations are now converging so that the term is stabilized in education to denote a sense of transcendence and compassion in the class that recognizes the interrelatedness of the teacher, students, and the subject matter (Tisdell, 2003).

### Spirituality is beyond religiosity

For a long time, the concept of spirituality has been interchangeably used with religiosity despite their critical differences. In a seminal study in this domain, Nash (2002) distinguished these two terms by contending that religion is a public institution that accelerates one’s access to a power greater than ourselves, while spirituality is a personal construct. In simple words, religion is what we do with others, whereas spirituality is what we do inside ourselves. According to Nash (2002), religion can be perceived as head, while spirituality is heart. Likewise, Syihabuddin (2017) differentiated religion from spirituality by arguing that the former emanates from one’s previous beliefs and teachings, while the latter is the practice of a belief. Syihabuddin also regarded spirituality as an inclusive life experience that is fundamental for a person’s life, while religion is more like a social institution, a belief, and a ritual practice. In sum, spirituality goes beyond religiosity and other similar concepts in the sense that it takes two directions into consideration; internal relationship with self as well as external relationship with others.

### Spiritual pedagogy as an approach

Throughout the long history of teaching and education, many approaches have been proposed and implemented in different contexts. As pinpointed by Shimabukuro (2008), such instructional approaches can be divided into three types, namely *transmissional*, *generative*, and *transformative* approaches. The first approach considers students as passive agents, simple receivers of materials, and the object of teaching (Johnson, 2010). Conversely, in this approach teachers are materials providers and presenters and the students have no option but to listen to what their teacher gives them or tells them to do (Johnson, 2010). The approach regards teachers as banks of knowledge and students and the consumers of knowledge. However, the generative approach moves forward and draws on theoretical underpinnings of constructivism, exploratorism, collaborativism, cooperativism, and discovery-learning. In so doing, it gives the students an active role in shaping their learning journey and constructing knowledge based on their own experiences and interactions with others (Wittrock, 1974; Fiorella and Mayer, 2016). The third approach expands the boundaries of education and pedagogy to include activities that help students construct a social-oriented knowledge in the classroom (Johnson, 2010). Based on the proponents of transformative approaches, an effective education should go beyond immediate contexts and concerns and pursue broader social issues. By this approach, students are inspired to critically think about the world and social problems existing in their societies. Therefore, learning is concurrently a personal and transformative process (Johnson, 2010).

Along with these three approaches, in the past decades, there has emerged a propensity towards pedagogical practices oriented to spiritual values, which came to be known as spiritual pedagogy (SP). In particular, SP as an approach to education and instruction reflects the ideas emphasized in humanistic psychology and affective pedagogy in that it encourages teacher to practice in tune with “values” that constitute the basis of their job including emotion, affection, humility, sensibility, sympathy, love, patience, tolerance, and the like (Llewellyn, 1998; Wright, 2003; Syihabuddin, 2017). A pedagogical approach based on spirituality can develop a millennial generation that constantly hones teachers’ and students’ knowledge and skills to fulfill the requirements of the contemporary world (Syihabuddin, 2017). Although this approach has been applied to counselling training, management, primary education, moral education, and science (Erricker and Erricker, 2000; Harlos, 2000; Wiersma, 2004; Delaney et al., 2007; Harris, 2007; Moss, 2011; Pettersen, 2015; Fowler et al., 2020), its implementation in L2 education is still in its initial stages and requires deep research.

### Features of a spiritual pedagogy

For implementing SP in an educational setting, some core features must be represented in teachers’ classroom practices and activities. The first feature has to do with teachers’ capability to understand students’ spirituality and experiences (Shimabukuro, 2008). They must be able to detect and interpret such experiences that determine students’ development. The second characteristic of SP is that the teachers take a contemplative attitude toward their pupils and constantly think of their future, knowledge, and how to lead them. This contemplation brings about creativity in teachers’ practices and students’ learning. Magnanimity is the next feature of SP which concerns being receptive to the presence of the spirit among people and the community. It also denotes the representation of generosity and benevolence towards different views in academia (Shimabukuro, 2008). The fourth feature pertains to teachers’ interpersonal awareness and ability in their job, especially during their interactions with students, colleagues, parents, and school principals. The fifth characteristic of SP concerns teachers’ use of various classroom activities and strategies to maintain their own teaching motivation as well as their students’ motivation to actively take part in their learning process. The last feature is teachers’ spiritual leadership, which can be developed by spiritual practices that penetrate into the teacher’s character (Syihabuddin, 2017).

### Research on spiritual pedagogy

After making numerous attempts to elucidate the concept of spirituality, scholars turned their attention toward exploring the role and potentials of SP in different fields in the past couple of decades. For instance, in Arts and Literature, Schiller (1999) ran classic study on the use of SP in improving students’ performance in these subjects by employing non-cognitive experiences that reflect students’ needs. Furthermore, in management, Harlos (2000) conducted an influential study that unraveled the definition of spirituality and its applications together with some practices to implement a SP in the context of management in New Zealand. She provided a common view of spirituality from which different personal meanings could be extracted that reflect teachers’ interpretations and instructional goals. She also experimentally indicated how SP can guide one’s teaching. In a similar manner, in the higher education context of the United States, Hochheimer (2013) conducted an experimental study on the power of SP in promoting undergraduate students’ learning throughout a semester. He gave students different task reflecting spiritual praxis and pedagogy and found that their learning, confidence, and spiritual exploration improved at the end of the course. In counselling training, Swinton (2016) ran an exploratory study on 8 counsellors over a period of 11 months to see the presence and impact of spirituality in their training programs. In the end, the researcher found spiritual awareness as a useful tool for generating a transformative learning and an engaging atmosphere for training. Additionally, correlational studies have been carried out that signify that spirituality is related to different variables such as positive mental health, wellbeing, hope, social functioning, and empathy (Berger, 2002; Doolittle and Farrell, 2004; Houskamp et al., 2004; Smith, 2006; McEntee et al., 2013; Mollaei et al., 2019). In contrast to other fields, researching spirituality and SP in ELT is taking its initial steps. As a case in point, in their seminal study, Mahboob and Courtney (2018) provided a general picture of SP in L2 education by arguing that spirituality of L2 teachers play a crucial role in their pedagogical practices, professionalism, identity construction, and students’ academic performance. They also maintained that the place of spirituality in teachers’ beliefs and practices is still an odd gap in ELT research and practice. To bridge the gap, they provided and sought out five broad questions that clarified how teachers’ spirituality can influence their professional practice. Likewise, Galeh and Dorcheh (2015) investigated the possible connection between language teaching and spirituality in Iran drawing on constructivist perspectives. After reviewing the literature, they found that pedagogy based on spirituality facilitates learning by enhancing sense of autonomy, self-confidence, empathy, self-esteem, and decreasing stress and anxiety. Moreover, in Indonesia, Masbur and Syahril (2021) ran a qualitative study on SP and offered a model to implement SP known as “Collaboration on Based Three Circle Components” (CoBa3CC), which had three forms; class-based SP, culture-based SP, and community-based SP. Despite these promising studies, the impact of SP on L2 education is still insufficiently explored. ELT is a fertile ground for SP as English language is an international language (Galeh and Dorcheh, 2015). Hence, L2 teachers and educators must take spirituality into account in order to establish modesty, humility, and worldviews among the stakeholders.

### The implementation of a spiritual pedagogy in L2 education: Practices and applications

Research approves that SP fosters students’ spiritual attitudes, cultural identity, proper social behaviors, morality, wellbeing, and functionality (McEntee et al., 2013; Mollaei et al., 2019; Fowler et al., 2020). In order to implement SP in the context of second/foreign language education, EFL teachers and practitioners are recommended to take advantage of some practices in their instruction as what follows: (1) using a personalized version of SP (e.g., asking the students to retell and reflect on their own experiences of spirituality and spiritual values to promote their participation and cooperation), (2) loving pedagogy is also a venue for integrating spirituality into L2 education as spirituality itself is a positive emotion (Palmer, 1993; Harlos, 2000; Smith et al., 2012; Barton and Miller, 2015; Wang et al., 2022). For instance, teachers can cultivate spiritual values and beliefs among their students after establishing a sense of love in the classroom. This professional love fosters SP, as well. (3) combining SP and PP, which are claimed to go hand in hand (Barton and Miller, 2015) to cultivate spirituality in their students (e.g., by enhancing students’ positivity and positive emotions, teachers can go beyond cognitive and linguistic layers of L2 education to the spiritual layer), (4) authorizing the students by shifting from a ‘banking’ form of knowledge transmission to an “engaged” pedagogy (e.g., practicing discovery learning approaches that involve spiritual values), (5) leading the class *via* teachers’ personal stories of spiritual values in their life and profession (e.g., by running discussion sessions and debates in the class), (6) establishing an affective engagement in the class (e.g., by reducing the distance between the teacher and students and forming a sense of compassion and interrelatedness), (7) constructing a space of allowance for diverse perspectives (e.g., having discussions in the class on different perspectives on specific spiritual values), and (8) implementing character-based education. This strategy is practical in that some students may come from different cultures with different values, hence they may be more spiritual than other. Teachers can recognize such character variations and highlight common spiritual values in line with them.

**Figure 1:**
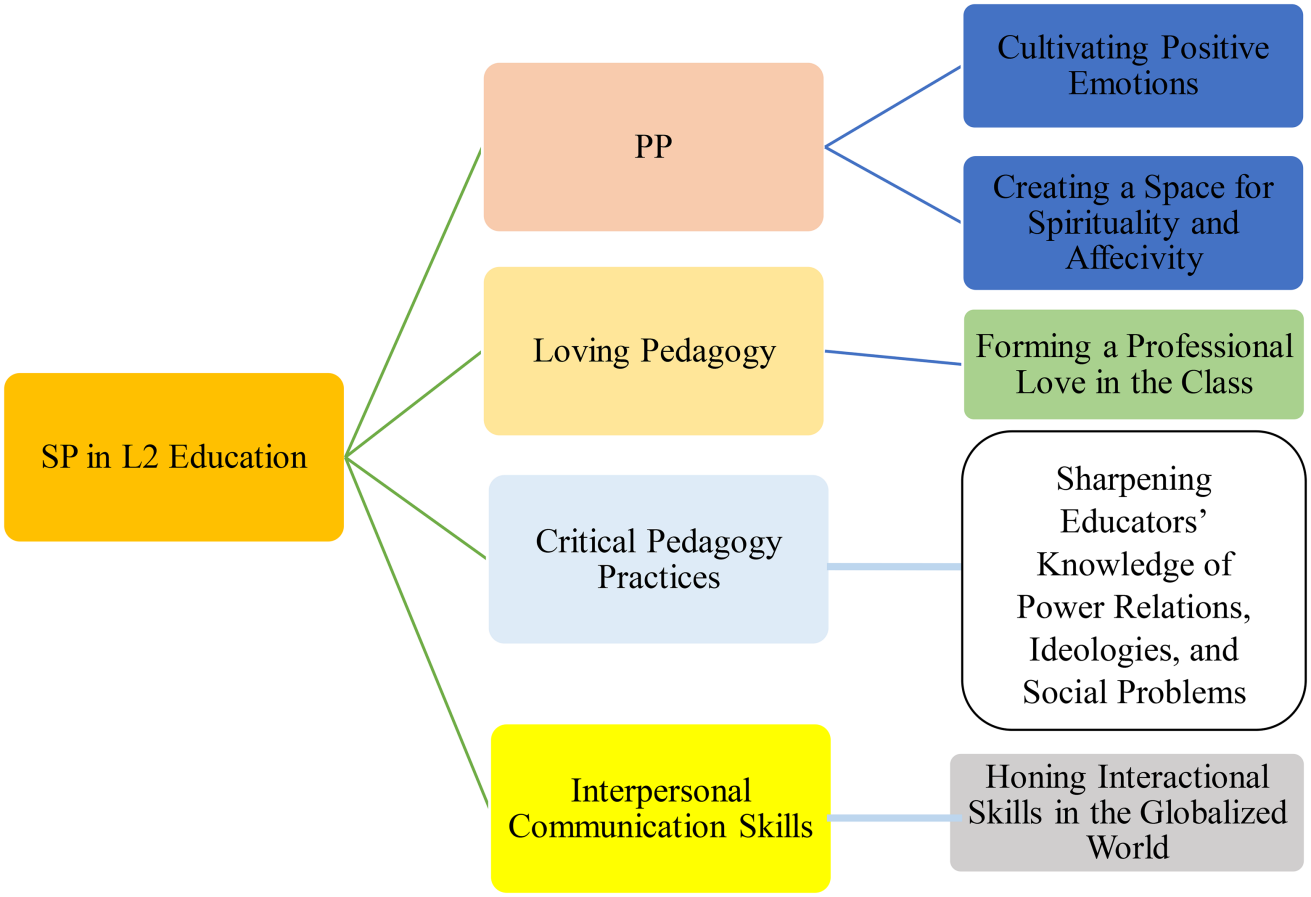
Practices for implementing SP in L2 education.

More specifically, quite consistent with the emotional contagion mechanisms of emotional transfer (see Shao et al., 2020), EFL teachers can use techniques such as explanation of spiritual issues, modeling, discussion, reflective practices, presentation, question and answer, group work, and oral reproduction of stories as some strategic approaches to implement SP in their classes. The scope of such pedagogical practices can range from personal, school, and community-based spiritual values to those at (macro) cultural levels. While the present study endorses the potentials of these practices for implementing SP in L2 education, it strongly commits itself to the belief that the integration of SP into loving pedagogy, PP, interpersonal communication skills, and practices of critical pedagogy (e.g., changing the hierarchy of student-teacher relationship, considering issues of power and control) can elucidate the role of SP in SLA more tangibly (Figure 1).

These four elements can be applied to real teaching practice by taking some steps and using some strategies. To implement PP, educators can explain and cultivate positive emotions like joy, love, happiness, resilience, optimism, stroke, immediacy, and many more in students, which foster spirituality. It can be asserted that spirituality and positivity go hand in hand and in case L2 classes enshrine these factors, a superior learning occurs. Moreover, loving pedagogy can be applied in L2 classes by creating a space of professional love in the class by caring for students’ emotions, needs, and expectations. When students feel loved, they may go beyond cognitive and linguistic concerns and seek spirituality in education. Critical pedagogy practices can also pave the way for SP in that using activities that sharpen educators’ knowledge and awareness of power relations, ideologies, and social problems extends education from immediate context to macro contexts and concerns. When students realize the criticality of sociocultural and spiritual values and beliefs, their desire for SP enhances, too. Finally, SP can be applied through developing interpersonal communication skills like credibility, clarity, confirmation, and so forth. When these skills are honed, educators’ concerns go beyond simple linguistic competencies. Instead, they quest for interpersonal communication in a globalized world in which they can share and discuss their cultural identity, spiritual values, and beliefs that require spiritual awareness.

## Concluding remarks

In this article, it was argued that SP is an effective tool for generating numerous positive outcomes in L2 education for both teachers and students. It was also maintained that spirituality and values are inseparable parts of EFL teachers’ and students’ identity and performance, hence the integration of a pedagogy based on spiritual values into L2 classes is suggested, especially in cultural contexts where English cannot convey the exact meanings and interpretations of a particular cultural value. For instance the interpretations of poems and literary works may be better captured in an educational context oriented toward spirituality. As a result, the present review article can be helpful for EFL teachers, students, teacher educators, curriculum designers, and L2 researchers. EFL teachers may find this study useful in that they can realize the power of spirituality and values in their pedagogical practices and behaviors in the classroom. EFL students can also benefit from this review study in that their awareness and sensitivity to spirituality increases. For them, learning is no longer a one-way street with the teacher being “the sole source of knowledge,” but a process connected to one’s spirit and inner emotions. Another group that can take advantage of this article is teacher trainers who mostly present their professional development courses with the intention of improving EFL teachers’ pedagogical practices without considering spirituality and teaching from heart. Hence, they can run workshops, seminars, and courses to practically show EFL teachers how to inject spirituality into their practices. Depending on students’ fields and cultural backgrounds, EFL teachers can be provided with teaching methods and techniques that facilitate the cultivation of spiritual values in learners. Furthermore, curriculum designers can use the ideas put in this article as a starting point for planning and offering specific courses on spirituality for EFL teachers and students. Finally, L2 researchers can benefit from this study in that they can conduct further empirical studies in the context of L2 education to see how a SP can be implemented and cultivated. As stated in this study, most of the existing studies on SP are from fields other than L2 education. Therefore, L2 scholars are suggested to explore the dimensions, conceptualizations, and practical ways of implementing this type of education in L2 contexts using different research instruments. Moreover, future studies are recommended to develop and validate scales for SP specialized for SLA. The correlation of SP with many positive emotions driven from PP is also an interesting line for research. Likewise, future studies can focus on the developmental paths that EFL teachers take to form their identity as L2 teachers. Finally, this strand of research lacks a comprehensive model for implementing SP, hence further research is suggested to take actions against this backdrop and develop a universal model for SP that is applicable to different fields and different settings.

## Author contributions

The author confirms being the sole contributor of this work and has approved it for publication.

## Funding

This research was supported by the 2022 Annual Research Project on Teacher Education Curriculum Reform of the Education Department of Henan Province (2022-JSJYYB-034), the 2021 Annual Humanities and Social Science of the Education Department of Henan Province (2021-ZZJH-333), and the Research Project of School of Continuous Education in Xinyang Normal University (2017).

## Conflict of interest

The author declares that the research was conducted in the absence of any commercial or financial relationships that could be construed as a potential conflict of interest.

## Publisher’s note

All claims expressed in this article are solely those of the authors and do not necessarily represent those of their affiliated organizations, or those of the publisher, the editors and the reviewers. Any product that may be evaluated in this article, or claim that may be made by its manufacturer, is not guaranteed or endorsed by the publisher.
